# A novel application of mark-recapture to examine behaviour associated with the online trade in elephant ivory

**DOI:** 10.7717/peerj.3048

**Published:** 2017-03-09

**Authors:** Lydia M. Yeo, Rachel S. McCrea, David L. Roberts

**Affiliations:** 1 Durrell Institute of Conservation and Ecology, School of Anthropology & Conservation, University of Kent, Canterbury, Kent, United Kingdom; 2 Statistical Ecology @ Kent, School of Mathematics, Statistics and Actuarial Science, University of Kent, Canterbury, Kent, United Kingdom; 3 Interdisciplinary Centre for Cyber Security Research, University of Kent, Canterbury, Kent, United Kingdom

**Keywords:** CITES, Elephas, Internet, eBay, Loxodonta, Wildlife trade, Capture-recapture

## Abstract

The illegal trade in elephant ivory is driving the unlawful killing of elephants such that populations are now suffering unsustainable reductions. The internet is increasingly being used as a platform to conduct illegal wildlife trade, including elephant ivory. As a globally accessible medium the internet is as highly attractive to those involved in the illegal trade as it is challenging to regulate. Characterising the online illegal wildlife (ivory) trade is complex, yet key to informing enforcement activities. We applied mark-recapture to investigate behaviour associated with the online trade in elephant ivory on eBay UK as a generalist online marketplace. Our results indicate that trade takes place via eBay UK, despite its policy prohibiting this, and that two distinct trading populations exist, characterised by the pattern of their ivory sales. We suggest these may represent a large number of occasional (or non-commercial) sellers and a smaller number of dedicated (or commercial) sellers. Directing resource towards reducing the volume of occasional sales, such as through education, would enable greater focus to be placed upon characterising the extent and value of the illegal, “commercial” online ivory trade. MRC has the potential to characterise the illegal trade in ivory and diverse wildlife commodities traded using various online platforms.

## Introduction

Globally, environmental crime, including the illegal wildlife trade, is estimated to be worth $91–258 billion p.a. (UNEP, 2016), making it the fourth most lucrative class of crime after the drugs trade, counterfeiting and human trafficking. Further, its value is estimated to have increased by 26% between 2014 and 2016 (UNEP, 2016). As a specific category of environmental (wildlife) crime, the illegal wildlife trade is estimated to be worth $7–23 billion per annum (UNEP, 2016).

Since 2012 there has been a growing momentum towards recognition of wildlife crime, including illegal wildlife trade, as a serious crime requiring a response commensurate with its gravity. A series of events at national, regional and global levels have taken place to further this aim including the UK Conference on Illegal Wildlife Trade in 2014 and subsequent conference in Kasane, Botswana in 2015. Both events produced statements of intent consolidating next steps for aligned, anti-illegal wildlife trade activities, i.e., the London Declaration, 2014 (Government of the United Kingdom, 2014) and the Kasane Statement (Government of the United Kingdom, 2015). At the United Nations Congress on Crime Prevention and Criminal Justice in Doha, Qatar in 2015 a landmark development was achieved in the first tabling of wildlife crime as a Congress agenda item and its inclusion within the Doha Declaration adopted at that Congress (UNODC, 2015a). Shortly afterwards, the first United Nations Resolution to recognise the illegal wildlife trade as one of the largest transnational criminal activities, comparable to trafficking in drugs, arms and people, was adopted by the United Nations General Assembly (UNGA, 2015). This signalled heightened political concern over the adverse impacts of poaching and the illegal wildlife trade upon species, ecosystems and local communities as well as the need to counteract these (UNODC, 2015b).

Globally, enforcement agencies such as the International Criminal Police Organization (ICPO-INTERPOL) recognise that a high proportion of wildlife crime, including trade, is carried out by organised criminal networks, attracted by the area’s typically low risk and high profit nature (ICPO-INTERPOL, 2015; also see Wittig, 2016). A principal impetus driving formal recognition of wildlife crime as a serious crime is the pressing need to improve the effectiveness of counter-measures (Challender & MacMillan, 2014).

African elephant populations, from which the majority of traded ivory is sourced, are suffering unsustainable reductions as a result of illegal killing to supply the ivory trade (UNEP et al., 2013; CITES, IUCN & TRAFFIC, 2013; Wittemyer et al., 2014; Chase et al., 2016). The rate of killing now exceeds the growth capacity of the species, placing the African elephant population in net decline (Wittemyer et al., 2014). Further, the ability of depleted populations to withstand additional stressors, such as habitat loss, or environmental effects resulting from climate change, is likely to be compromised (Brook, Sodhi & Bradshaw, 2008; Barnosky et al., 2011).

The past two decades have seen a rapid increase in the online trade in wildlife, both legal, and illegal (IFAW, 2005; Beardsley, 2007; IFAW, 2008; Izzo, 2010; Shirey & Lamberti, 2011; Lavorgna, 2014). Since the Internet extends globally and is both readily accessible and challenging to regulate it has the potential to attract both legal and illegal traders. Research indicates that the Internet is being used as a medium to conduct illegal trade in wildlife (Magalhães & São-Pedro, 2012; Alves, Lima & Araujo, 2013; Lavorgna, 2014) with adverse impacts upon traded species (IFAW, 2005; IFAW, 2008; Izzo, 2010; Shirey & Lamberti, 2011). It is widely acknowledged that there is a need for effective means to address the threat to biodiversity posed by Internet-mediated illegal wildlife trade (Wyler & Sheikh, 2008; Bennett, 2011; Felbab-Brown, 2011; Shirey & Lamberti, 2011; ICPO-INTERPOL & IFAW, 2013; ICPO-INTERPOL & IFAW, 2014).

The Internet is a conduit for a significant volume of trade in elephant ivory, including illegal trade (IFAW, 2008; IFAW, 2011; ICPO-INTERPOL & IFAW, 2013). In response to lobbying, online trading sites, such as eBay, have banned the sale of ivory. However, the trade still continues (IFAW, 2008; IFAW, 2011; ICPO-INTERPOL & IFAW, 2013). Research into the online trade in ivory is needed to determine the scale of the problem and monitor activity; however, thisis challenging. Since the word “ivory” describes a colour, as well as an organic material, online searches for ivory items will result in postings for ivory coloured items, and also items made from ivory. The number of postings for ivory coloured items (e.g., curtains, rugs and furniture) tends to far exceed that for items made from ivory, making the latter difficult to distinguish within the overall trading volume. In addition to this linguistic camouflage derived from using the word ‘ivory’, deliberate devices, such as describing elephant ivory as a physically similar but legitimately traded material (e.g., horn or bone) may be used to actively conceal illicit postings (Harrison, Roberts & Hernandez-Castro, 2016). Consequently, the process of detecting online elephant ivory postings is complex and implicitly resource-intensive. Further, since law enforcement officers are currently unable to monitor internet sites continuously, and check every item they detect for sale, they are likely to detect only a fraction of the illegally traded ivory that is actually for sale (ICPO-INTERPOL & IFAW, 2013; Hernandez-Castro & Roberts, 2015). Accurate knowledge of the extent of the illegal trade and the traders involved is key to informing and prioritizing intervention activities to curb illegal trade. Research indicates that anticipated shifts in the preferred Internet medium for illegal wildlife trade from the open, or surface, web to the so-called “dark web” have not, so far, occurred (Harrison, Roberts & Hernandez-Castro, 2016). This, coupled with the increasing volume of illegal wildlife trade conducted via the surface web, suggests that it remains an attractive medium for illegal trade and may indicate a lack of effective enforcement measures applied to counter this trade (Harrison, Roberts & Hernandez-Castro, 2016). Therefore, using current monitoring techniques the observed trade is likely to only represent the tip of the iceberg. Statistical methods are therefore required to provide an understanding of the trading population.

Mark-recapture (MRC) has been applied in a range of fields to estimate total population size (Böhning, 2008) and/or to estimate demographic parameters of interest (Lebreton et al., 1992; Amstrup, McDonald & Manly, 2005) from an observed sample. Its use in estimating the size of cryptic, including criminal, populations has been recognised in a number of sociological contexts; illicit drug use or supply (Bouchard, 2007; Vaissade & Legleye, 2009), the illegal drug and arms trades (Bloor, 2005) and estimation of victim numbers from terrorism (Murphy, 2009). As far as we are aware MRC has, however, never been used to investigate illegal online trades, although others (e.g., Lavorgna, 2015; Vida et al., 2016) have attempted to provide an understanding of the dynamics. In this paper we apply MRC to a novel situation to explore behaviours associated with the illegal, online trade in elephant ivory conducted via eBay UK. Specifically, we employ three different marks, i.e., item number, item description (or title) and seller username (or “ID”), to explore demographic parameters of interest that may be indicative of illicit trading in terms of detection probability.

## Methods

### Data specification and acquisition

The study design was approved by the University of Kent, School of Anthropology and Conservation’s Research and Ethics Committee. We defined a single data point as an advertisement (posting) on eBay UK for an item for sale within the UK which results from the search terms of “Ivory; Antique; UK only”. For each item, we recorded its Description (Title), Item Number and Seller Identification details (username). This information was recorded once per week for an eight-week period, starting on 28th March 2014. Data were collected on the same weekday and at approximately the same time of day (i.e., Fridays at 10.30 a.m. ± 30 min). Prior research based on an intensive survey had identified this data collection period as the weekly peak in the number of items of interest being posted and therefore represented an optimal sampling window (Yeo, 2011).

### Data assessment: identification of ivory items

Two former law enforcement experts examined postings to assess whether items comprised, or contained, ivory and, if so, the likely origin of that ivory (i.e., its category). We defined categories of ivory as: elephant, hippo, walrus, ox/cow-bone, man-made, other or unknown. A period of approximately 8 h was allowed for assessment of each week’s set of recorded data, to reflect a standard working day; in essence the two experts replicated their previous roles in wildlife enforcement in terms of sifting items that would be taken forward for subsequent investigation. This period delimited the total number of items which could be assessed from each of the eight (Friday) downloads. It should be noted that prior to the experts being provided the list of items, item records were randomised to prevent bias associated with how the eBay “Relevance” sort function generates the order of search results.

### Data analysis: open population model

Encounter history matrices consisting of 1s, denoting captures, and 0s, denoting non-captures, were constructed for each of three categories of data associated with the downloaded elephant ivory postings,

1. Description: the title description of the posted item, which was assumed to be unique per item due to the low probability of using the same words in the same order.
2. Item (number): the unique item number associated with each posting, and
3. Seller: the unique user identification name associated with each posting.

An open population mark-recapture model was fitted to these data. The model used was the POPAN form of the Jolly–Seber (JS) model (Schwarz & Arnason, 1996) which permits the estimation of population size whilst allowing the population to be open. Nineteen models within the JS framework were fitted to the encounter history matrices. The models encompassed constant (.), time-dependent (*t*) or heterogeneous (*h*) variants of the parameters listed here,

*N*: population size,

*p*: capture probability,

*β*: probability of arrival in the population,

*ϕ*: retention (or survival) probability.

Heterogeneity was modelled as a mixture of two binomials such that proportion *π* of the population has the capture probability of *p*1 and proportion (1 − *π*) has the capture probability of *p*2. Closed population models were considered as constrained versions of the JS model. Models with no new arrivals are denoted by *β*(=1) and models with no departures are denoted by *ϕ*(=1). ΔAIC was used as a tool for model selection and applied to rank the models (Burnham & Anderson, 2002). When appropriate, we used AIC weights to produce model-averaged estimates which account for model uncertainty.

In order to investigate the heterogeneity within capture probability further, the open population Cormack–Jolly–Seber (CJS) model (which conditions on the first capture and hence does not allow estimation of population size) was fitted to the Sellers data to assess the significance of an individual covariate—the average number of items associated with each Seller over the study period. This allowed investigation of whether capture probability (*p*) was significantly related to the number of items an individual has for sale (Table 1). The individual covariate could not be incorporated into the JS model due to the unseen individuals having unknown values of individual covariate (see chapter 7 of McCrea & Morgan, 2014).

**Table 1:** Open population Cormack–Jolly–Seber mark-recapture model: covariate model selection.

Model	ΔAIC	*k*
*ϕ*(.), *p*(covariate)	0.00	3
*ϕ*(.) *p*(.)	22.65	2
*ϕ*(.) *p*(*t*)	30.49	8
*ϕ*(*t*)*p*(.)	33.00	8
*ϕ*(*t*) *p*(*t*)	39.68	13

**Notes.**

*ϕ* retention (or “survival”) probability (.) constant *p* probability of capture logit (*p*) *α*_0+_*α*_1_ × covariate (covariate) individual covariate, i.e., average number of items for sale (*t*) time dependent *k* number of parameters

## Results

**Figure 1:**
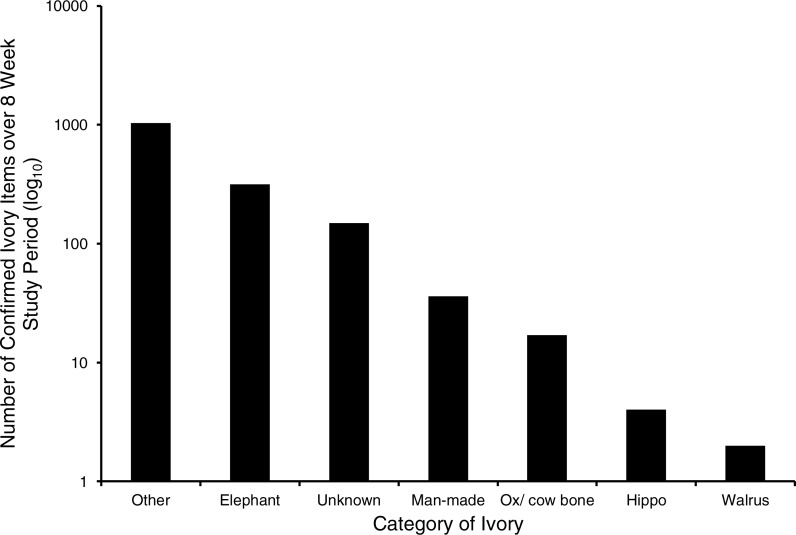
Histogram illustrating absolute and relative amounts of categorised ivory items identified by visual assessment of online postings over the eight week study period (unique values only).

Between 528 and 633 postings were recorded from eBay UK, per week, for the eight-week study period. Between 349 and 419 (c. 68%) of these postings were randomised and then assessed each week by two third party enforcement experts, according to how many items could be assessed in approximately 8 h. In total 7% were found to concern elephant ivory, which equated to 42–67 items per week, based on Item Number. Elephant ivory items ranked second after “Other” items (i.e., non-material ivory items), however it should be noted that “Unknown” items (i.e., those that could not be identified) ranked third (Fig. 1).

Table 2 presents the top 10 models (as ranked by ΔAIC) for each of the data sets. It is clear that the models incorporating capture heterogeneity, *p*(*h*), are strongly supported by the sellers and descriptions data sets (ΔAIC to next best model of 19.42 and 21.67 respectively). There was no evidence of capture probability heterogeneity from the Items data set and the ΔAIC of the top-ranked models are much closer. AIC weights showing the relative plausibility of each of the models are displayed for the item data. These weights were used to produce model-averaged estimates which are displayed in Table 3. There was no support for the closed population models for any of the three data sets.

**Table 2:** Open population mark-recapture POPAN form of the Jolly–Seber model: model ranking and selection using ΔAIC.

Sellers	*k*	ΔAIC	Items	*k*	ΔAIC	AIC weight	Descriptions	*k*	ΔAIC
*N*(.), *β*(.), *p*(*h*), *ϕ*(.)	6	0.00	*N*(.), *β*(.), *p*(.), *ϕ*(*t*)	10	0.00	0.59	*N*(.), *β*(.), *p*(*h*), *ϕ*(.)	6	0.00
*N*(.), *β*(.), *p*(.), *ϕ* (.)	4	19.42	*N*(.), *β*(*t*), *p*(.), *ϕ*(*t*)	16	1.02	0.35	*N*(.), *β*(.), *p*(.), *ϕ*(.)	4	21.67
*N*(.), *β*(*t*), *p*(.), *ϕ*(.)	10	24.45	*N*(.), *β*(*t*), *p*(*t*), *ϕ*(.)	17	5.81	0.03	*N*(.), *β*(.), *p*(.), *ϕ*(*t*)	10	26.51
*N*(.), *β*(.), *p*(*t*), *ϕ*(.)	11	25.69	*N*(.), *β*(.), *p*(*t*), *ϕ*(*t*)	17	7.54	0.01	*N*(.), *β*(.), *p*(*t*), *ϕ*(.)	11	28.56
*N*(.), *β*(.), *p*(.), *ϕ*(*t*)	10	29.30	*N*(.), *β*(.), *p*(.), *ϕ*(.)	4	8.91	0.01	*N*(.), *β*(*t*), *p*(.), *ϕ*(.)	10	29.17
*N*(.), *β*(*t*), *p*(*t*), *ϕ*(.)	17	33.98	*N*(.), *β*(*t*), *p*(*t*), *ϕ*(*t*)	23	9.66	0.00	*N*(.), *β*(*t*), *p*(.), *ϕ*(*t*)	16	35.83
*N*(.), *β*(*t*), *p*(.), *ϕ*(*t*)	16	34.67	*N*(.), *β*(*t*), *p*(.), *ϕ*(.)	10	10.62	0.00	*N*(.), *β*(.), *p*(*t*), *ϕ*(*t*)	17	37.38
*N*(.), *β*(.), *p*(*t*), *ϕ*(*t*)	17	35.74	*N*(.), *β*(.), p(h), *ϕ*(.)	6	12.91	0.00	*N*(.), *β*(*t*), *p*(*t*), *ϕ*(.)	17	38.05
*N*(.), *β*(*t*), *p*(*t*), *ϕ*(*t*)	23	44.66	*N*(.), *β*(.), *p*(*t*), *ϕ*(.)	11	15.12	0.00	*N*(.), *β*(*t*), *p*(*t*), *ϕ*(*t*)	23	47.95
*N*(.), *β*(=1), *p*(*t*), *ϕ*(.)	10	60.50	*N*(.), *β*(*t*), *p*(*t*), *ϕ*(=1)	16	177.70	0.00	*N*(.), *β*(.), *p*(*t*), *ϕ*(=1)	10	82.75

**Notes.**

*N* population size (.) constant (*t*) time dependent (*h*) heterogeneity *p* capture probability *β* probability of arrival in the population *ϕ* retention (or “survival”) probability *k* number of parameters ΔAIC measure of each model relative to model of best fit by AIC

**Table 3:** Open population mark-recapture POPAN form of Jolly–Seber model: maximum likelihood estimates (MLE) and corresponding standard errors (SE). Note that the MLEs for the items data are model-averaged estimates from the top two models as ranked by AIC.

Parameter	Sellers MLE (SE)		Items MLE (SE)		Descriptions MLE (SE)	
*β*_1_	0.67	(0.10)	0.10	(0.02)	0.31	(0.05)
*β*_2_	–	–	0.13	(0.02)		
*β*_3_	–	–	0.11	(0.01)		
*β*_4_	–	–	0.13	(0.02)		
B_5_	–	–	0.13	(0.02)		
*β*_6_	–	–	0.11	(0.02)		
*β*_7_	–	–	0.13	(0.02)		
*p*1	0.54	(0.05)	0.77	(0.07)	0.06	(0.02)
*p*2	0.02	(0.04)	–		0.58	(0.08)
*π*	0.09	(0.13)	1^a^		0.95	(0.02)
*ϕ*_1_	0.88	(0.03)	0.35	(0.08)	0.74	(0.05)
*ϕ*_2_	–	–	0.38	(0.09)	–	–
*ϕ*_3_	–	–	0.24	(0.06)	–	–
*ϕ*_4_	–	–	0.30	(0.07)	–	–
*ϕ*_5_	–	–	0.45	(0.08)	–	–
*ϕ*_6_	–	–	0.47	(0.08)	–	–
*ϕ*_7_	–	–	0.11	(0.05)	–	–
*N*	710.00	(1125.00)	360	(27.11)	1614.00	(539.00)

**Notes.**

*π* proportion of individuals with capture probability *p*1^a^ *β* probability of arrival in the population *p* capture probability *ϕ*_*j*_ time-dependent retention (or “survival”) probability *N* population size

a *π* is not estimated in the case of no heterogeneity.

The maximum-likelihood estimates from the sellers and descriptions analysis (Table 3) indicate the existence of two groups of individuals with markedly different capture probabilities. Proportion 0.09 of the sellers population has capture probability 0.54, whilst the remaining proportion of the population has capture probability 0.02. The model-averaged estimates for the items data demonstrate some suggestion of time-dependence in both arrival and retention probabilities, and this may be linked to items being re-posted with a new item number during the study.

AIC model selection from fitting the CJS model to the sellers data strongly supported the model with capture probability depending on the average number of items a seller has listed (Table 1). The maximum-likelihood estimates (Table 4) indicate that as the number of items a seller has listed increases, so does the probability of capture of an individual seller (*α*1 = 0.68, SE =0.19).

**Table 4:** Maximum likelihood estimates (on the logistic scale), corresponding standard errors and 95% confidence limits from fitting the Cormack–Jolly–Seber model to the Sellers data.

Parameters	MLE	SE	Lower 95% point	Upper 95% point
*ϕ* (survival)	1.43	0.22	0.99	1.86
*α*_0_ (intercept)	−1.34	0.34	−2.01	−0.66
*α*_1_ (slope)	0.68	0.19	0.30	1.06

**Notes.**

*ϕ* retention (or “survival”) probability *α*_0_ intercept in logistic regression *α*_1_ coefficient of covariate value in logistic regression

It is clear from Table 3 that the estimates of population size, *N*, are estimated with very poor precision and therefore it is impossible to draw any conclusions from them. It is known that when capture probability is very low it is very difficult to obtain meaningful estimates of population size (see for example p. 45 of McCrea & Morgan, 2014). If capture probability of the less detectable population could be increased then the precision of estimates might improve, however such an approach would require greater resources for identifying occasional sellers of illegal ivory online.

## Discussion

Our results indicate that an online trade in elephant ivory is being conducted via eBay UK, despite the existence of eBay’s User Agreement and Animal and Wildlife Products Policy (AWPP) policies and in contravention of these. Under its AWPP (eBay, 2015a), described by eBay as reflective of international trade restrictions and treaties banning the sale of ivory, eBay prohibits the sale of ivory with the limited exception of antiques that contain 5% or less of real ivory and were made before the year 1900. None of the elephant ivory postings we identified complied with the terms of the AWPP, so all constituted prohibited sales. Further, sellers were acting in contravention of eBay’s User Agreement (eBay, 2015b), which is framed as a contractual arrangement and stipulates seller responsibilities, including compliance with eBay’s Prohibited and Restricted Items Policy (eBay, 2015c) and AWPP.

**Figure 2:**
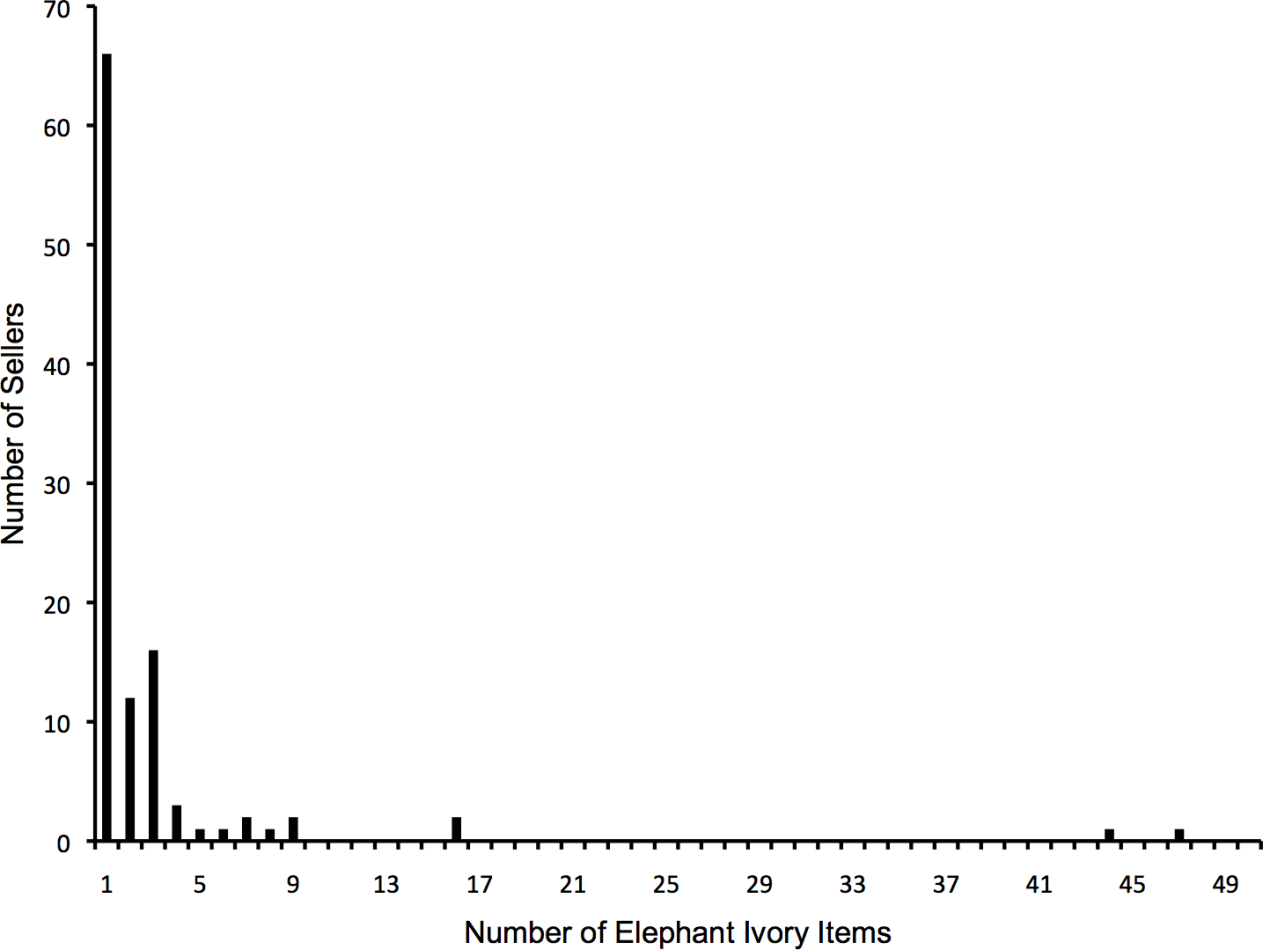
Histogram illustrating the number of observed, confirmed elephant ivory items for sale per observed seller during the 8-week study period.

According to our categorisation of the types of ivory traded, elephant ivory forms the second largest group after “Other” (i.e., ivory coloured items such as textiles and furniture) (Fig. 1). Our data infer that two elephant ivory trading populations are active on eBay UK characterised by their trading patterns and associated capture probabilities. One, inferred population has a relatively high capture probability (*p* = 0.54, SE 0.05) whereas the other population has a relatively low capture probability (p = 0.02, SE 0.04). Capture probability is, as one would expect, positively related to the number of items that a seller has listed (i.e., the more items that a seller has listed, the more “catchable” they are). The population with a relatively high capture probability sells elephant ivory persistently, and tends to have multiple items advertised for sale simultaneously. This trading pattern may be suggestive of dedicated (or commercial) sellers. In contrast, the population with a relatively low capture probability tends to sell elephant ivory items only occasionally, and as single items. We suggest that this sporadic, lower-volume sales pattern may be associated with occasional (or non-commercial) sellers. Relatively few sellers are trading persistently in multiple items of ivory, with high catchability, and a comparatively large number of sellers are trading sporadically and typically in single items, with low catchability (Fig. 2). However it is worth noting that items categorised as “Unknown” (i.e., those that could not be identified) ranked third (Fig. 1) and therefore creates a level of uncertainty as one would expect when making decisions based solely on the available online attributes of an item.

Catchability may also vary according to whether a seller is using the “Auction” or “Buy it Now” option to sell items. Items posted using “Buy it Now” are likely to have a longer residence time and are therefore more likely to be detected. Such items may be associated with dedicated (commercial) sellers, trading in larger volumes of ivory. In contrast, items posted for sale using the “Auction” facility are likely to have a shorter residence time, making them more difficult to detect. Items posted in this way may be associated with the “less catchable”, occasional (non-commercial) sellers. Such differences in residence time may also be seen in the use of item descriptions and item number as ‘marks’ during the mark-recapture analysis. We suggest that using “Description”, rather than Item Number would provide a more robust “mark” for future studies. We found that the latter changes during relisting of an unsold item, whereas we found “Description” largely remained unchanged except in a few cases where “New listing” was added to the item description.

The existence of the two, inferred populations has implications beyond the suggestion of two potential classes of online (i.e., eBay UK) ivory seller. The observed pattern of relatively few, persistent, higher-volume sellers and relatively numerous, occasional, lower-volume sellers leads to the high standard errors reported for population size (*N*) (Table 3).

The pattern of high number of single offence, versus individuals engaged in multiple offences is likely to be seen in other area of environmental crime. This makes it difficult for law enforcers both to estimate the total population of offenders, and to identify the most persistent offenders from within this population. Since one would imagine that a lower likelihood of prosecution is associated with single offences, resource-driven priorities mean that enforcement focus tends to be directed towards multiple offenders. This has two effects: firstly, a single offender population tends to persist and, secondly, the relatively high volume of single versus multiple offenders complicates overall (offender) population size estimation. However, if law enforcers are mainly interested in persistent offenders then an analysis focusing on individuals with a high probability of capture may be of interest and may result in a more robust estimate of this specific population.

We see potential opportunities, resulting from this study, for actions to address the online trade in elephant ivory. For example, should the many, sporadic, single items sales actually be associated with occasional sellers, then this might indicate a lack of understanding of trading requirements, rather than deliberate offending; it would be interesting to see whether these individuals use code words such as “ox bone” often used to disguise the sale of elephant ivory (Harrison, Roberts & Hernandez-Castro, 2016) which may indicate a level of intent. If it is a case of lack of understanding on the part of the sporadic, single item sellers, education to raise awareness and understanding of legal and policy requirements surrounding the trade in ivory may be of value. Should compliance subsequently increase, then the twin benefits of a reduction in the volume of sporadic, online trade and a lessening of the confounding effect of this trading pattern upon overall trading population size (and value) estimation may result. In our example, reducing the number of sporadic, less catchable sellers should allow more focus to be placed upon detection and characterisation of persistent, higher volume sellers. Reduction of the high standard errors associated with the sporadic seller trading pattern should better enable estimation of the total, online trading population size, and its monetary value. Such evidence may assist enforcement agencies in directing their resources towards persistent, higher volume sellers with a greater potential for successful prosecution. However, absolute estimates of the size of the market activity may not be required, as relative size may be adequate if prioritizing worst offenders or monitoring the efficacy of schemes to reduce offending rates is the goal.

An assessment of the speed with which elephant ivory postings appear, and then disappear from eBay UK prior to this study yielded very few with a residence time on the site of ≤1 h (Yeo, 2011). Posting items very briefly is sometimes used as a mechanism by those engaged in illegal trade to highlight the availability of illegal items but avoid detection by the authorities. However, the fact that we did not detect evidence for this phenomenon does not mean that offline discussion of posted ivory items to conclude sales is not occurring. We have been informed that this phenomenon has also been seen in China (Anon, pers. comm.); however, there the items are reposted under a different username. This activity was not observed in this study.

Our research was confined to the eBay UK online market, for transactions taking place within the UK, and indicates that MRC may be applied to gain a clearer understanding of the online ivory trade when sampling is not continuous. MRC exhibits potential for scaled up research into the online ivory trade across a wider area of cyberspace. Further, since trade in elephant ivory takes place via other electronic (social) media, MRC may also offer a means to research and characterise trade conducted via those media and the degree to which trading platforms overlap.

Research into the electronically-mediated wildlife trade is still in its infancy with the number of peer-reviewed studies slowly increasing. At the same time, the pressing need for enhanced understanding of its key characteristics, especially to elucidate illegal trade, is a conservation priority. The illegal, online trade in wildlife commodities, including elephant ivory, is a serious and growing issue that presents a significant conservation threat. Despite a groundswell in international intent to stem the illegal wildlife trade there will, necessarily, be a time lag between planning and execution of impactful intervention measures. Further, it is unlikely, given the desire for items of wildlife that demand for them will disappear in the near-term (Courchamp et al., 2006; Hinsley, Veríssimo & Roberts, 2015). Given this, the application of MRC offers a flexible and resource-efficient means by which to assess, more accurately, key facets of the illegal online trade in wildlife, as well as other criminal activity (see Wittig, 2016). The enhanced understanding that this approach brings may usefully inform regulatory and intervention measures to support delivery of wider conservation imperatives.

## Additional Information and Declarations

### Data Availability

The following information was supplied regarding data availability: The raw data was provided for peer review but, due to the risk of the data being de-identified and exposing the individuals studied, it cannot be published with the article.
